# Comparison of Marginal Adaptation of MTA and CEM Cement Apical Plugs in Three Different Media

**DOI:** 10.22037/iej.2016.15

**Published:** 2016

**Authors:** Fatemeh Ayatollahi, Mahdi Tabrizizadeh, Fatemeh Zare Bidoki, Reza Ayatollahi, Milad Hazeri Baqdad Abad

**Affiliations:** a *Department of Endodontics, Dental School, Shahid Sadoughi University of Medical Sciences, Yazd, Iran; *; b * Department of Pediatric Dentistry, Dental School, Shahid Sadoughi University of Medical Sciences, Yazd, Iran; *; c * Dental School, Shahid Sadoughi University of Medical Sciences, Yazd, Iran; *; d * Department of Orthodontics Dentistry, Dental School, Shahid Sadoughi University of Medical Sciences, Yazd, Iran *

**Keywords:** Calcium-Enriched Mixture, Marginal Adaptation, Mineral Trioxide Aggregate

## Abstract

**Introduction:**

The aim of this study was to compare the marginal adaptation of mineral trioxide aggregate (MTA) and calcium-enriched mixture(CEM) cement apical plugs in open apex teeth in dry, blood- and saliva-contaminated canals.

**Methods and Materials:**

A total of 120 human extracted single-rooted teeth were used. The teeth were decoronated and canals were cleaned and shaped up to #80 K-Files. After simulating open apex condition, samples were randomly divided into two groups (Group 1: MTA plug and group 2 CEM cement plug) and each group was then further divided into 3 subgroups (dry canal, blood- and saliva-contaminated canals). MTA and CEM cement apical plugs were placed into the canal. After full setting of apical plug, immediate marginal adaptation of the samples was assessed by electronic microscope. The data were analyzed using the two-way analysis of variance.

**Results:**

There was no statistically difference between the average marginal gap in MTA and CEM cement groups in three different conditions. The average gap in dry canal was significantly lower than canal contaminated with blood and saliva.

**Conclusion:**

It seems that marginal adaptations of MTA and CEM apical plug was not significantly different in various conditions and the two materials can be used successfully in this method.

## Introduction

The root canal treatment of immature teeth with open apices is considered as a challenge for dentists. Because of the wide apical region and lack of apical constriction, the procedures of working length determination, gutta-percha compaction and avoiding extrusion of material from the apical region are very difficult [1-3].

Different methods such as the use of artificial apical barrier and apexification with long-term application of calcium hydroxide are used for the treatment of such teeth. Today, the use of artificial apical plug is preferred to traditional method of apexification due to its advantages such as the less treatment sessions, short time of treatment session and less dependence on patient’s cooperation. In this method, a plug made of materials such as mineral trioxide aggregate (MTA), calcium-enriched mixture (CEM) cement, calcium hydroxide or absorbable ceramics, is placed in the canals as apical barrier [4-8].

MTA is one of the commonly used materials in the apical plug method due to its good sealing ability, biocompatibility and proper antibacterial properties [4-6]. On the other hand, CEM cement has comparable, if not better sealing ability and biocompatibility in comparison with MTA. On the other hand, the higher antibacterial properties and lower setting time are considered as its advantages over MTA [6, 9-13].

Numerous studies have shown the importance of leakage in endodontic treatment failure [14]. The proper material selection with high sealing ability is necessary for apical plug creation [6, 15]. The correlation between microleakage and marginal adaptation was shown in studies by Shani *et al*. [16] and Torabinejad *et al*. [17]. Canal contamination with blood, saliva and moisture during obturation can influence the properties of the MTA and CEM cement [18-20].

The aim of this *in vitro *study was to compare the marginal adaptation of MTA and CEM cement apical plugs in open apex teeth dry, blood- and saliva-contaminated canals.

## Materials and Methods

This experimental *in vitro *study was conducted on 120 single-rooted and single-canal human teeth. After disinfection of the teeth (1 h immersion in 5.25% NaOCl solution), the teeth were then kept in normal saline [21]. The Crowns were cut by diamond disc (010, Tizkavan, Tehran, Iran) to prepare a standardized 13-mm root length from the apex. By Using a K-File #15 (Dentsply Maillefer, Ballaigues, Switzerland), the working length was determined. The apical part of canals were cleaned by step back method using #15-40 K-Files and then the canals were enlarged to #80. Canals were irrigated between each instruments use with 2.25% NaOCl. In the next step, the tips of #1-4 Peeso drills (Dentsply Maillefer, Ballaigues, Switzerland) were passed orthogradely into the apical foramen to simulate the conditions of the open apex teeth. Finally, the size of apical foramen was 1.3 mm. The specimens were assayed under stereomicroscope (Carl Zeiss, f170, Germany) under 25× magnification to ensure the absence of cracks had in the samples [22].

Canals were filled with 1 mL of 17% ethylenediaminetetraacetic acid (EDTA, Ariadent, Tehran, Iran) for 3 min and then were rinsed by 5 mL of normal saline. Samples were randomly divided into two groups: group 1, MTA apical plugs and group 2 CEM cement plugs. Also each group was divided into 3 subgroups (dry canal, saliva-contaminated and blood-contaminated). 

Group 1A: Angelus MTA (Angelus, Londrina, PR, Brazil) was prepared according to the manufacturer's instructions and after drying the canals, it was placed into the canals by the MTA carrier (Dentsply Maillefer, Ballaigues, Switzerland) and it was condensed with #3 and 4 hand pluggers (Dentsply Maillefer, Ballaigues, Switzerland) in such a way that ultimately the apical plug with a thickness of 5 mm was created. A small sponge was used to avoid MTA extrusion. Thickness and density of apical plug was assessed by radiographic method. After placement of a wet paper into the canal, the access cavity was filled by temporary restoration (Coltosol; Ariadent, Tehran, Iran). The teeth were kept for 24 h in an incubator at 37^°^C with 100% humidity. After 24 h, the temporary restorations were removed, the setting of MTA was checked and then the remained canal space was filled with gutta-percha (Diadent, Chongju, Korea) and AH-26 sealer (Dentsply DeTrey, Konstanz, Germany) using lateral compaction technique.

Group 1B: Venous blood was taken from one of the volunteers (who had signed an informed consent) and was inserted into the tube containing 50 IU heparin (Alborzdarou, Tehran, Iran) per mL of blood. In this group, canal spaces were filled with blood and then were aspirated precisely in which only the walls were contaminated with blood. Other steps were the same as the procedures in group 1A. 

Group 1C: In this group, immediately before the study, the salvia of a staff was collected and the canal spaces were filled with it. The additional salvia was aspirated precisely in which only the walls were contaminated with saliva. Other steps were performed as same as the procedures in groups 1A and 1B. 

In the next three sub-groups (2 A, 2B, 2C) all steps were the same as MTA group, with the difference being that, CEM Cement (BioniqueDent, Tehran, Iran) was used for placement of apical plug.

All the teeth were stored for 24 h in normal saline. One-mm-thick cross-sections one- and two-mm away from the apex were cut by diamond disc. Samples were prepared for assessment under a scanning electronic microscope (SEM) (TESCAN VEGA3, Czesh Republic) and then the gap between the canal walls and plug was evaluated by microscope under 600× magnifications. The surface of each sample was divided into 4 parts equally and the maximum width of the gap between the plug and the canal wall in each section was recorded.

Finally, the data were analyzed using the two-way analysis of variance using SPSS software (SPSS version 20, SPSS, Chicago, IL, USA).

## Results

Table 1 shows the mean (SD) of the gap diameter between the plug and the canal wall in the experimental groups.

The average gap diameter in dry, blood-contaminated and salvia-contaminated canals in the MTA group was higher than CEM cement group, although the difference was not statistically significant. As shown in Table1, the average gap diameter in dry canals (4.260±0.274) was significantly less than canals contaminated with blood (5.515±0.287) and saliva (5.022±0.285) (*P*=0.007). 

**Table 1:** Mean (standard) deviation of marginal gap (µm

	Dry	Blood	Saliva
MTA	4.329 (0.382)	5.749 (0.392)	4.979 (0.403)
CEM cement	4.192 (0.392)	5.280 (0.403)	5.065 (0.403)
*P*-value	1.000	0.960	1.000

## Discussion

The aim of this study was to compare the marginal adaptation of MTA and CEM cement apical plugs in dry, blood-contaminated and saliva-contaminated canals. Investigating the marginal adaptation provides the indirect evaluation of sealing ability of materials used in the root canal obturation. The studies confirmed that there is a direct link between marginal adaptation and sealing ability [16, 17, 23-25].

For creation of open apex conditions, in different studies, Peeso Reamer and sulfuric acid [26] have been used. Since, root absorption created by sulfuric acid caused some irregularity in canal walls and may have an influence on the gap diameter between the plug and the wall, in this study the Peeso Reamer was used [26].

Some studies showed that the smear layer has an adverse effect on marginal adaptation of endodontic materials. Marginal adaptation of MTA was significantly increased when 17% EDTA was used [27]. In order to remove the smear layer and increase marginal adaptation, 17% EDTA was used in the present study [21, 27, 28].

In order to investigate the gap diameter between the apical plug and canal walls, SEM was used. This microscope has some advantages in comparison with optical microscope such as higher resolution. On the other hand, this method is more common, affordable and more accessible compared to micro-computed tomography (micro-CT) method. It is reported that higher intake of electronic microscopes can cause cracks in the hard tissues [23]. Since in this study, cracks play a confounding role, during the experiments, the samples were investigated by stereomicroscope for several times during the observation. Samples with cracks were replaced by samples without any cracks.

To ensure the width measurement of gap, the surface of each sample was divided into 4 equal parts and the maximum width of the gap between the plug and the canal wall was recorded for each part. 

The results of this study indicated that although the average gap in three different environments in the MTA group was higher than CEM cement group, this difference was not statistically significant. Bolhari *et al. *[22] showed that there was not a significant difference between the marginal adaptation of MTA and CEM cement and root-end cavity walls which is contaminated with blood and saliva. The results of his study was in contrast with our study. 

It should be noted that based on the results of this research, the average gap generally in dry canal is lower than the canal contaminated with saliva and blood. Studies have shown that the physical properties of MTA and CEM cement will be reduced by blood contamination, which could be due to the effects of the crystal formation [22, 29-33]. The same reason could justify the results of this study. However, due to complex clinical conditions, the question how much of gap can decreases the success rate of treatment, remains to be answered with further clinical studies.

## Conclusion

According to the results of the present study, the marginal adaptation of MTA and CEM cement apical plugs with canal walls was not significantly different in the various conditions. Both materials showed favorable results in this method, although, the contamination of canal with blood and saliva decreases the marginal adaptation of both materials within the canal walls. Due to the advantages of the apical plug technique, further clinical research is recommended.
